# BTG1 might be employed as a biomarker for carcinogenesis and a target for gene therapy in colorectal cancers

**DOI:** 10.18632/oncotarget.10649

**Published:** 2016-07-18

**Authors:** Shuang Zhao, Shu-rui Chen, Xue-feng Yang, Dao-fu Shen, Yasuo Takano, Rong-jian Su, Hua-chuan Zheng

**Affiliations:** ^1^ Cancer Center, Key Laboratory of Brain and Spinal Cord Injury of Liaoning Province, and Animal Center, The First Affiliated Hospital of Jinzhou Medical University, Jinzhou, China; ^2^ Department of Science and Technology, Jinzhou Medical University, Jinzhou, China; ^3^ School of Health Science, Tokyo University of Technology, Nishi-Kamata, Ohta-ku, Tokyo, Japan; ^4^ Life Science Institute of Jinzhou Medical University, Jinzhou, China

**Keywords:** colorectal cancer, BTG1, carcinogenesis, aggressive phenotypes, gene therapy

## Abstract

Here, BTG1 overexpression inhibited proliferation, induced differentiation, autophagy, and apoptosis in colorectal cancer cells (p_2_ arrest might be related to Cyclin B1 and Cdc25B hypoexpression in HCT-15 transfectants, while G_1_ arrest in HCT-116 transfectants overexpressing p21 and p27. BTG1 overexpression decreased the expression of Bcl-2, Bcl-xL, XIAP, Akt1 or survivin and increased the expression of Bax or p53 in colorectal cancer cells. BTG1-induced autophagy was dependent on Beclin-1 expression. BTG1 overexpression might weaken β-catenin pathway in colorectal cancer cells. The chemosensitivity of BTG1 transfectants to paclitaxel, cisplatin, MG132 or SAHA was positively correlated with its apoptotic induction. There was a lower expression level of BTG1 in cancer than matched non-neoplastic mucosa by RT-PCR (p

## INTRODUCTION

Colorectal cancer is the third most common cancer in both men and women, accounting for nearly 10% of new cases in all cancers. Incidence rates have been decreasing for most of the past two decades, which has been attributed to the alteration in risk factors and the selection of colorectal cancer among adults 50 years and older [1, 2]. Pathomolecular observations demonstrate that colorectal carcinogenesis undergoes the malignant transformation of adenoma-adenocarcinoma, but its molecular mechanisms remain elusive.

BTG (B-cell translocation gene) family includes six proteins (BTG1, BTG2, BTG3, BTG4, Transducer of ErbB-2, and TOB2), which suppresse proliferation, cell cycle progression and induce differentiation as a tumor suppressor [3, 4]. The anti-proliferation activity of BTG proteins is mediated via Caf1a and Caf1b deadenylase subunits of Ccr4-NOT complex [5]. BTG1 was originally identified in B-cell chronic lymphocytic leukemia and considered as a potential biomarker to monitor the complete remission of acute myeloid leukemia [6]. The N-terminal domain of BTG1 bears an LxxLL motif favoring nuclear accumulation, and another region encompassing Box A inhibiting nuclear localization [7]. C-terminal region of BTG1 interacts with the nuclear receptor TRα and the myogenic factor MyoD [8]. Additionally, BTG1 binds to protein arginine methyltransferase 1 *via* Box C region [9]. Human carbon catabolite repressor protein-associative factor 1 can interact with BTG1, which depends on the phosphorylation of p34cdc2/Cyclin E and p34CDK2/Cyclin A kinase site on BTG1 ser-159 [10].

BTG1 expression is the highest in G_0_/G_1_ phases of cell cycle and decreases the progression of cells through G_1_ phase [11]. BTG1 was reported to enhance Hoxb9-mediated transcription and suppress proliferation of HeLa cells [12]. BTG1 was localized to the apoptotic cells with appearance of DNA fragmentation and nuclear condensation, in line with the BTG1-mediated apoptosis of NIH 3T3 cells [13]. Further study shows that BTG1 functions as Bcl-2-regulated mediator and is involved in antisense Bcl-2-mediated cytotoxic effects in breast cancer cells [14]. BTG1 overexpression may suppress the proliferation of myoblasts and induce their differentiation [15]. The down-regulated expression of BTG1 was detected in both lung and breast cancers [16, 17]. Our previous work has shown that BTG1 overexpression inhibited proliferation, migration, invasion, tumor growth, lung metastasis, and induced G_2_/M arrest, differentiation, senescence, apoptosis and chemosensitivity in gastric cancer cells. BTG1 hypoexpression was observed in gastric cancer and negatively correlated with depth of invasion, lymphatic and venous invasion, lymph node metastasis, TNM staging and worse prognosis of gastric cancer [18]. To clarify the roles of BTG1 in colorectal carcinogenesis, the expression of *BTG1* mRNA and protein was investigated in colorectal cancer. In addition, the effects of BTG1 overexpression on aggressive phenotypes of colorectal cancer cells were analyzed with phenotype-related molecules examined. Finally, the in vivo effects of BTG1 overexpression on tumor growth of colorectal cancer cells were assessed in nude mice.

## RESULTS

### The effects of BTG1 overexpression on proliferation and cell cycle of colorectal cancer cells

**Figure 1:**
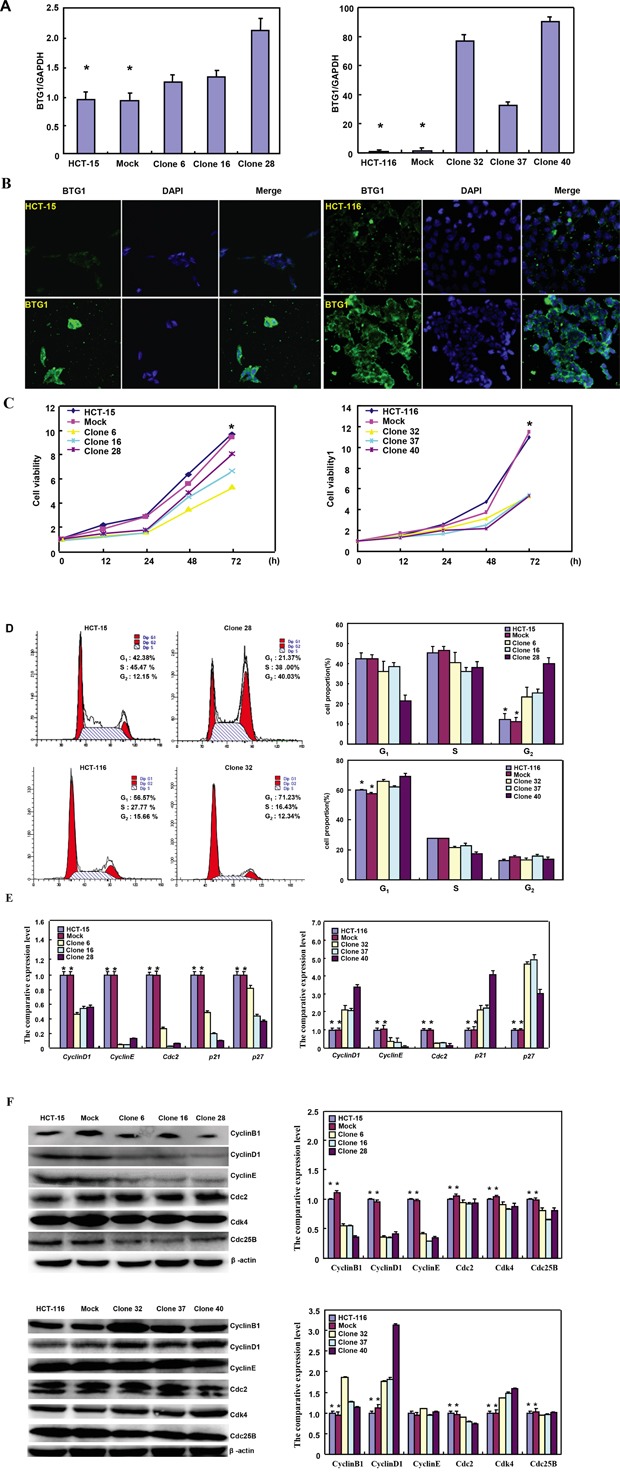
The effects of BTG1 overexpression on proliferation and cell cycle of colorectal cancer cells After transfection of pcDNA3.1-*BTG1*, BTG1 expression became strong in HCT-15 and HCT-116 cells by RT-PCR **A**., and immunofluorescence **B**. The transfectants showed a lower growth in comparison to the control and mock **C**. Ectopic BTG1 expression could induce G_2_ arrest of HCT-15 transfectants by PI staining, while G_1_ arrest of HCT-116 transfectants **D**. The cell-cycle- related molecules were screened by real-time RT-PCR **E**. and Western blot **F**. *, *p* < 0.05, compared with the transfectants.

To clarify the roles of *BTG1*, its expressing plasmid was successfully transfected in HCT-15 and HCT-116 cells, evidenced by RT-PCR (Figure 1A), and immunofluorescence (Figure 1B). The transfectants showed a lower growth than the control and mock (Figure 1C, p<0.05). BTG1 overexpression caused G_2_ arrest in HCT-15 cells (*p*<0.05), while G_1_ arrest in HCT-116 cells (*p*<0.05) by PI staining (Figure 1D). BTG1 transfectants of HCT-15 showed the reduced expression of Cyclin D1, Cyclin E, Cdc2, p21 and p27, compared with the control and mock by real-time PCR (*p*<0.05, Figure 1E). Regarding HCT-116, BTG1 overexpression decreased the expression of Cyclin E and Cdc2 (*p*<0.05), but increased the expression of Cyclin D1, p21 and p27, compared with the control and mock (*p*<0.05, Figure 1E). At the protein level (Figure 1F), BTG1 overexpression decreased the expression of Cyclin B1, Cyclin D1, Cdc2, Cyclin E, Cdk4 and Cdc25B in HCT-15 cells (*p*<0.05). BTG1 overexpression down-regulated the expression of Cdc2 and Cdc25B (*p*<0.05), but up-regulated the expression of Cyclin B1, Cyclin D1 and Cdk4 in HCT-116 cells (*p*<0.05, Figure 1F).

### The effects of BTG1 overexpression on differentiation and autophagy of colorectal cancer cells

**Figure 2:**
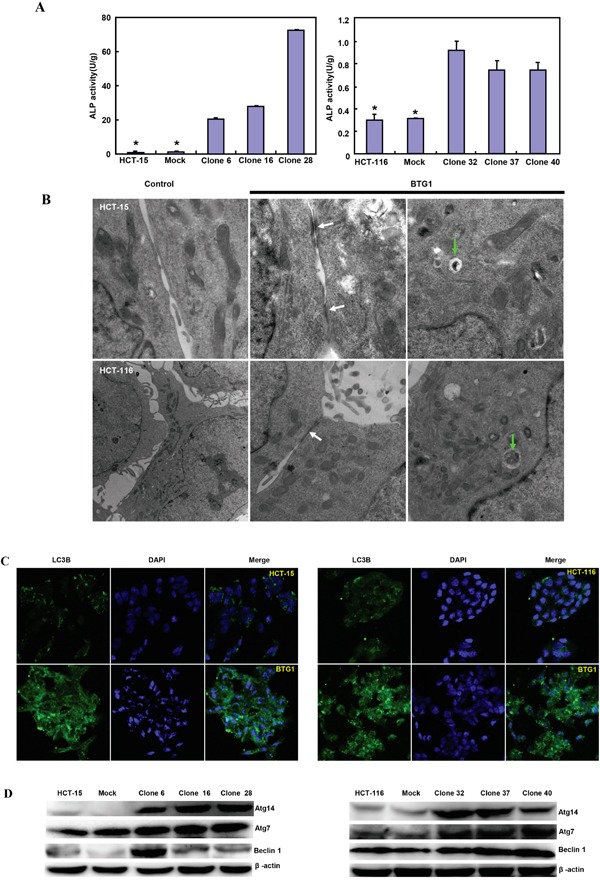
The effects of BTG1 overexpression on differentiation and autophagy of colorectal cancer cells BTG1 transfectants showed better differentiation, evidenced by alkaline phosphatase activity **A**. and tight junction under transmission electron microscope (TEM, B, white arrow). There was a high autophagy evidenced by autophagosome under TEM (**B**., green arrow) and LC-3B expression **C**. in BTG1 transfectants, compared with the control. Higher expression of Beclin-1, Atg7 and Atg14 was observed in BTG1 transfectants than the mock and control **D**. *, *p*<0.05, compared with the transfectants.

There was a better differentiation, evidenced by alkaline phosphatase activity (Figure 2A, p<0.05) and tight junction from transmission electron microscopy than the control (Figure 2B). A higher autophagy was evidenced by autophagosomes (Figure 2B) and LC-3B expression (Figure 2C) in both BTG1 transfectants than the control. At the protein level, BTG1 overexpression increased the expression of Atg7, Atg14 and Beclin-1 in both transfectants, compared with the control and mock (Figure 2D).

### The effects of BTG1 overexpression on apoptosis and senescence of colorectal cancer cells

**Figure 3:**
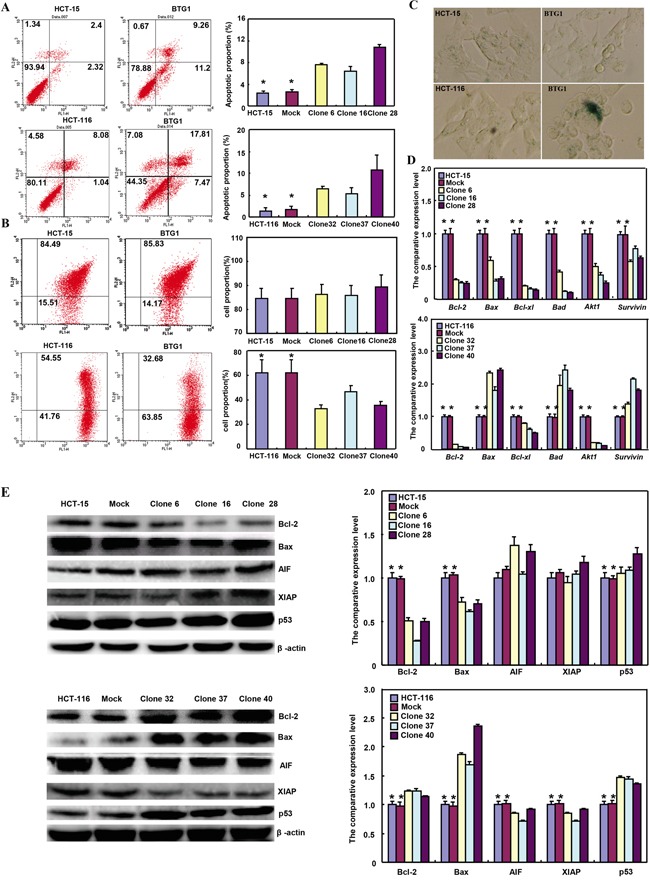
The effects of BTG1 overexpression on apoptosis and senescence of colorectal cancer cells Compared with mock and control, there was a higher apoptosis, evidenced by Annexin V assay **A**. in both transfectants. However, only HCT-116 transfectants showed a lower mitochondrial potential **B**. and a stronger β-galactosidase staining **C**. than the control or mock. The apoptosis-related molecules were screened by real-time RT-PCR **D**. and Western blot **E**. *, *p*<0.05, compared with the transfectants.

There was a higher apoptosis, evidenced by Annexin V-FITC staining in both BTG1 transfectants than the control and mock (Figure 3A, p<0.05). A lower mitochondrial membrane potential by JC-1 staining and a higher senescence by β-galactosidase staining were detectable in HCT-116 transfectants than the mock and control (Figure 3B and 3C, p<0.05), while there was no significant alteration in HCT-15 transfectants (Figure 3B and 3C, p>0.05). As shown in Figure 3D, BTG1 transfectants of HCT-15 showed reduced expression of *Bcl-2*, *Bax*, *Bcl-xL*, *Bad*, *Akt1* and *survivin*, compared with the control and mock by real-time PCR (*p*<0.05). In HCT-116, BTG1 overexpression decreased the expression of *Bcl-2*, *Bcl-xL* and *Akt1* (*p*<0.05), but increased the expression of *Bax*, *Bad* and *survivin*, compared with the control and mock (*p*<0.05). At the protein level (Figure 3E), BTG1 overexpression increased the p53 expression (*p*<0.05), but decreased the expression of Bcl-2 and Bax in HCT-15 transfectants (*p*<0.05). BTG1 overexpression down-regulated the expression of AIF and XIAP (*p* <0.05), but up-regulated the expression of Bcl-2, Bax and p53 in HCT-116 transfectants (*p* <0.05).

### BTG1 overexpression weakens β-catenin and p38 signal pathway in colorectal cancer cells

**Figure 4:**
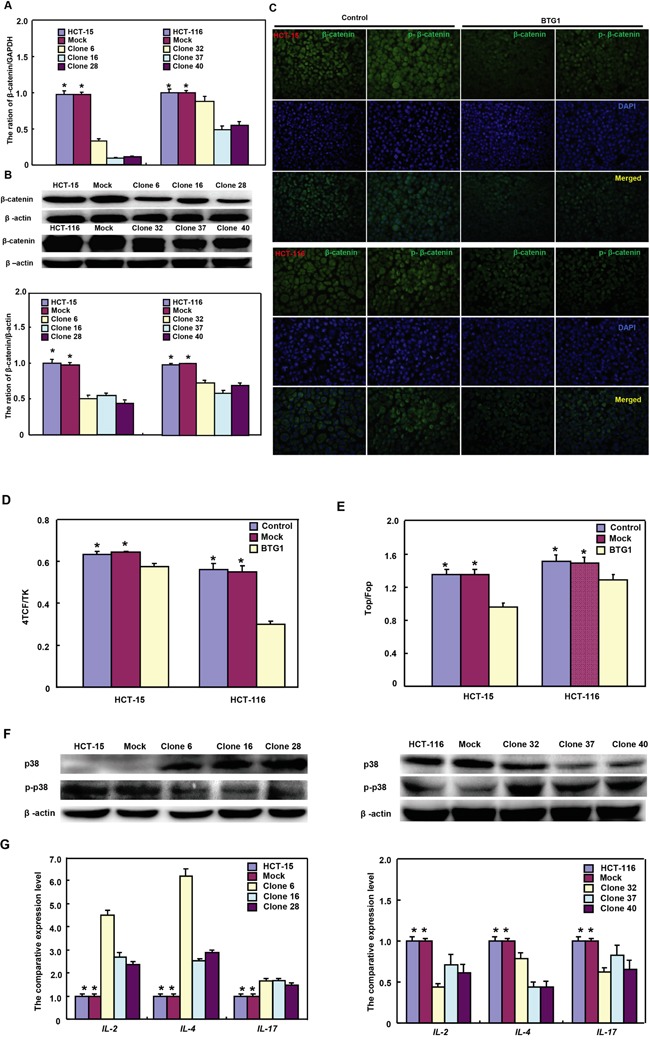
BTG1 expression targets β-catenin and phospho-p38 signal pathway in colorectal cancer cells After transfected with pcDNA3.1-*BTG1*, both HCT-15 and HCT-116 cells expressed less β-catenin mRNA and protein than the mock and control by RT-PCR **A**. and Western blot **B**. Immunofluorescence showed a lower expression of β-catenin and phosphorylated β-catenin in BTG1 transfectants than the control **C**. There was a lower activity of TCF-4 promoter **D**. and TCF-4-mediated transcription **E**. in BTG1 transfectants than the control by dual luciferase assay. According to Western blot **F**., higher p38 expression was seen in HCT-15 transfectants than the control, while the converse for phosphorylated p38 (p-p38). In HCT-116 cells, BTG1 transfectants showed lower p38 expression and higher p-p38 than the control. The target genes of p38 were screened by real-time RT-PCR **G**. *, *p* < 0.05, compared with the transfectants.

In HCT-15 and HCT-116 cells, both β-catenin expression and phosphorylation were decreased (Figure 4A-4C, p<0.05). Dual luciferase gene assay demonstrated that BTG1 overexpression might suppress TCF-4 promoter activity and TCF-4-mediated gene transcription activity (Figure 4D and 4E, p >0.05). Although p38 overexpression was detectable in HCT-15 transfectants, phospho-p38 protein showed down-regulated expression with a higher expression of its target genes, including *IL*-2, -4 and -17 (Figure 4F and 4G, p<0.05). However, it was the converse for HCT-116 transfectants (Figure 4F and 4G, p<0.05).

### The correlation between BTG1 expression and the sensitivity of colorectal cancer cells to chemotherapeutic agents

**Figure 5:**
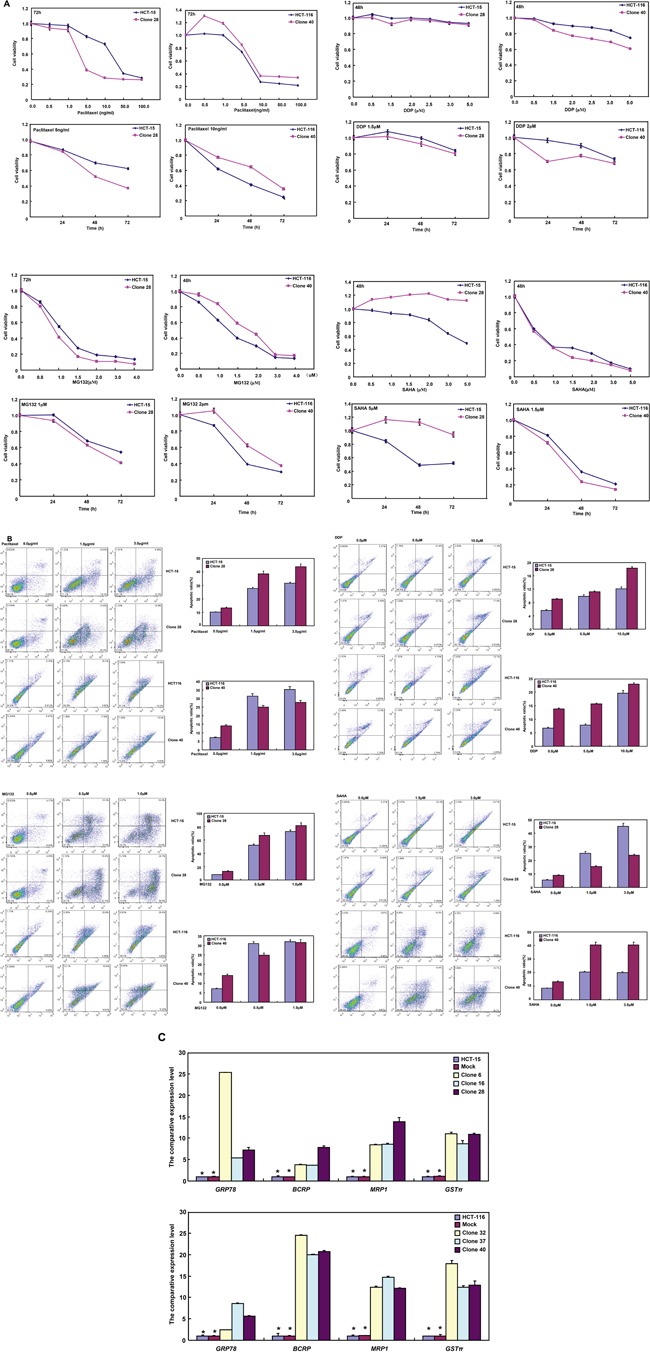
The relationship between BTG1 overexpression and chemosensitivity of colorectal cancer cells HCT-15 transfectant showed both higher sensitivity to paclitaxel, DDP and MG132 than the control and higher level of apoptotic induction **A** and **B**., but the converse for SAHA (A and B). HCT-116 transfectant was more sensible to DDP and SAHA, and had a higher apoptotic rate than the control (A and B), while versa for paclitaxel and MG132 (A and B). There appeared the mRNA overexpression of *GRP78*, *BCRP*, *MRP1* and *GST-π* in comparison to the mock and control by real-time RT-PCR **C**. *, *p* < 0.05, compared with BTG1 the transfectants.

After exposed to paclitaxel, cisplatin (DDP), and MG132, HCT-15 transfectants showed lower viability and higher apoptosis than the control in both time- and dose-dependent manners (Figure 5A and 5B, p <0.05), while the converse for SAHA. After treatment with DDP and SAHA, lower viability and higher apoptosis were observed in HCT-116 transfectants than the control in both time- and dose-dependent manners (Figure 5A and 5B, p <0.05), while the converse for paclitaxel and MG132. In addition, we found that mRNA expression of GRP78, BCRP, MRP1 and GST-π was up-regulated in both transfectants, compared with the control and mock (Figure 5C, p <0.05).

### BTG1 expression in colorectal cancers

**Figure 6:**
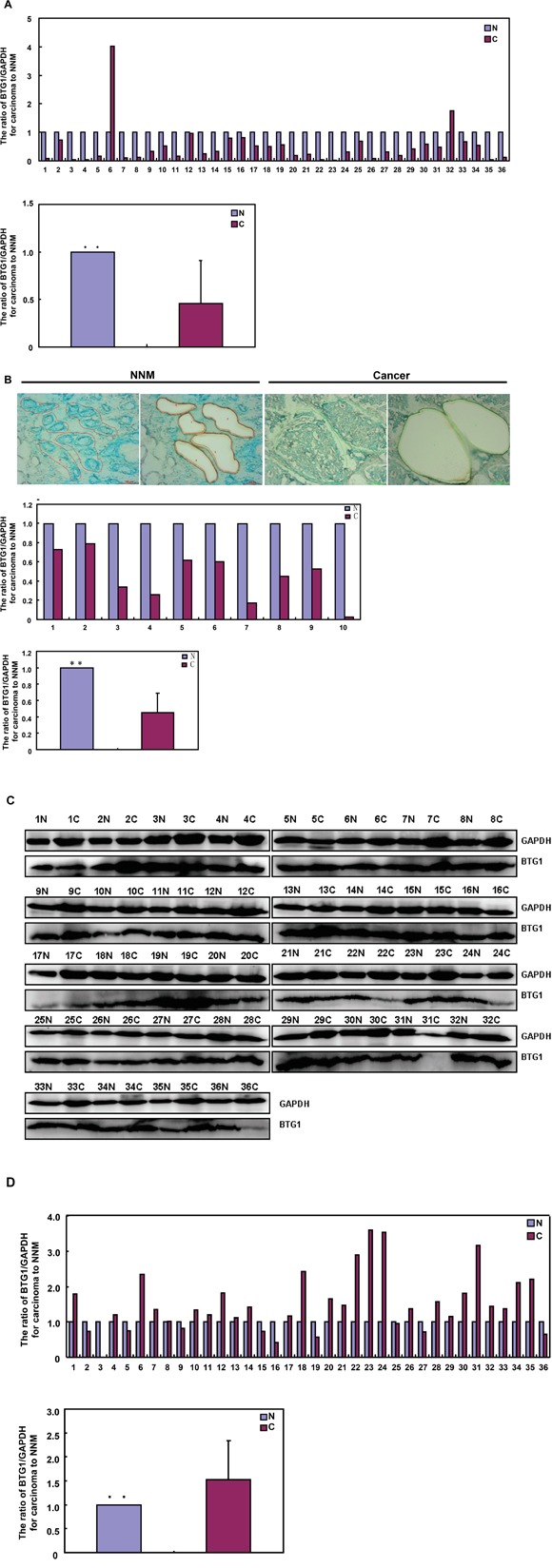
BTG1 expression in colorectal cancer and corresponding non-neoplastic mucosa *BTG1* was amplified by real-time RT-PCR with *GAPDH* as an internal control and quantitative analysis showed a lower *BTG1* mRNA expression in colorectal cancer than matched non-neoplastic mucosa (NNM, **A** and **B**). The colorectal gland and cancer cells were captured for real-time PCR. There was a lower expression of *BTG1* mRNA in colorectal cancer cells than normal glands (p<0.05, **B**). Additionally, there was more BTG1 expression (19 kDa) in the tissue lysates of colorectal cancer than paired NNM with GAPDH (37 kDa) as an internal control by Western blot and densitometry analysis **C** and **D**. N: non-neoplastic mucosa; C: cancer; **, *p* < 0.01.

Only 5.6% (2/36) cases showed *BTG1* mRNA overexpression in colorectal cancer, compared with matched mucosa. Statistically, the *BTG1* mRNA expression was decreased in colorectal cancer, in comparison with paired non-neoplastic mucosa (NNM, Figure 6A, p<0.05). To verify the results, we employed laser capture microdissection (LCM) to capture colorectal normal gland and cancer cells in 10 cases of CRCs and matched NNM. It was the same for real-time PCR (Figure 6B, p<0.05). Among 36 colorectal cancers, 25 cancers overexpressed 19kDa BTG1 protein in comparison to NNM. Densitometry analysis indicated that BTG1 protein was more detected in colorectal cancer than that in NNM (Figure 6C and 6D, p<0.05).

**Figure 7:**
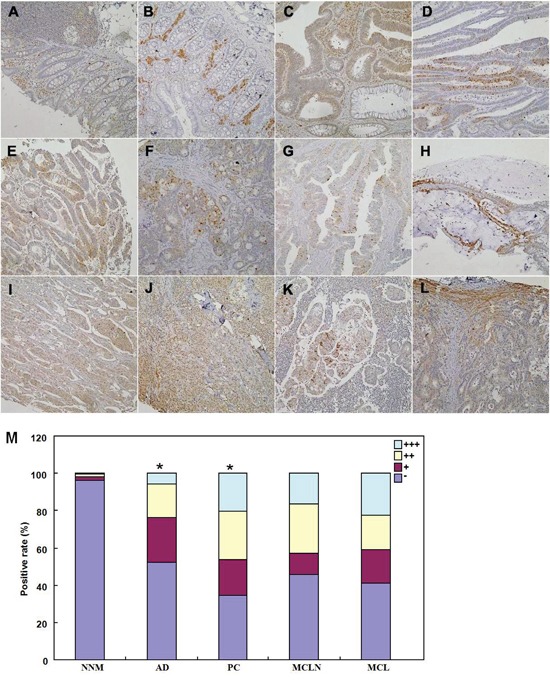
Immunohistochemical staining of BTG1 during colorectal carcinogenesis BTG1 protein was positively detected in the cytoplasm of colorectal mucosal epithelium **A, B**., infiltrating inflammatory cells (A), macrophages (B), lymphoid follicle (A), adenoma **C, D**., well-differentiated **E**., moderately-differentiated **F, G**., mucinous **H**. and poorly-differentiated **I, J**. adenocarcinoma, metastatic cancers in lymph node **K**. and liver **L**., and hepatocytes (L). There was higher BTG1 expression in adenoma and primary cancer than the non-neoplastic mucosa **M**. *, *p*<0.001; NNM, non-neoplastic mucosa; AD, adenoma, PC, primary cancer; MCLN, metastatic cancer in lymph node; MCL, metastatic cancer in liver.

As shown in Figure 7, BTG1 protein was distributed to the cytoplasm. BTG1 protein was positively detected in colorectal mucosal epithelium, infiltrating inflammatory cells, macrophages, lymphoid follicle, adenoma, well-, moderately- and poorly-differentiated, and mucinous adenocarcinoma, metastatic cancers in lymph node and liver. BTG1 expression was detectable in colorectal non-neoplastic mucosa (NNM, 3.8%, 18/475), adenoma (47.7%, 52/109), primary cancers (65.6%, 318/485), and metastatic cancers in lymph node (54.4%, 80/147) and liver (59.1%, 13/22), respectively. According to its frequency and density, BTG1 expression was statistically higher in colorectal cancer and adenoma than adjacent NNM (Figure 7M, p<0.05).

### The inhibitory effects of BTG1 overexpression on the tumor growth of colorectal cancer cells in nude mice

**Figure 8:**
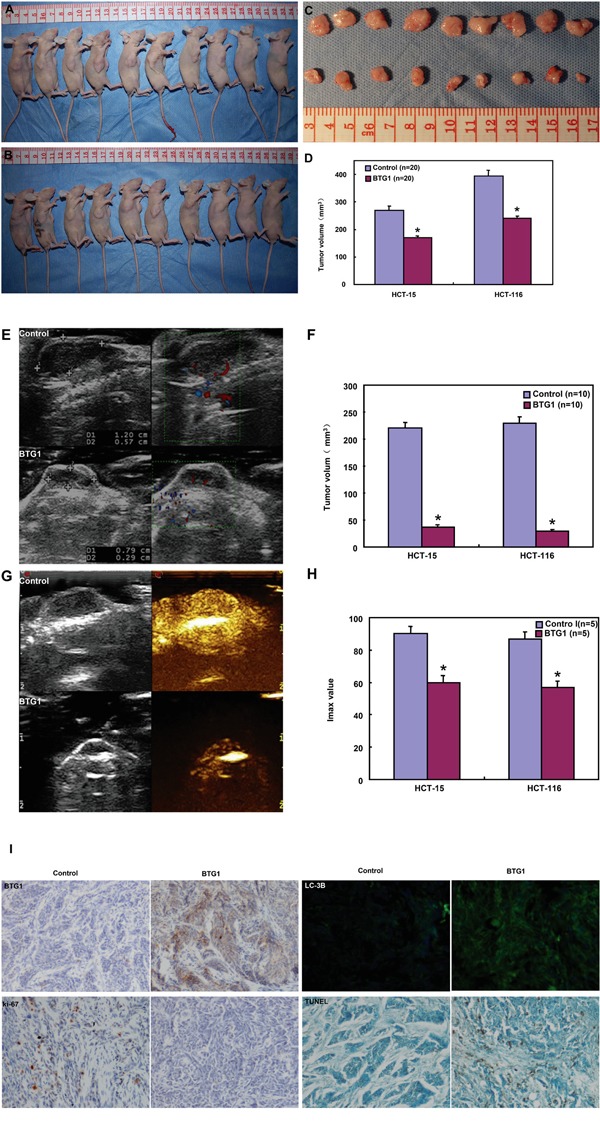
BTG1 overexpression suppresses the growth of colorectal cancer cells in nude mice The growth of both HCT-15 and HCT-116 cells were faster than BTG1 tranfectants by measuring tumor volume **A-D**. and ultrasonic imaging **E, F**. The tumor displayed a lower blood supply than the control by contrast-enhanced ultrasonic imaging **G, H**. There appeared stronger BTG1 expression in transfectant tumors than the control by immunohistochemistry **I**. The transfectant tumors showed weaker Ki-67 expression, higher signals of TUNEL and higher LC-3B immunoreactivity than the control (**I**). *, *p*< 0.05, compared with BTG1 transfectant the control; Imax, maximum intensity.

HCT-15, HCT-116 and their transfectants were subcutaneously transplanted into immune- deficient mice. The tumor volumes of both HCT-15 and HCT-116 cells xenografts were larger than those of BTG1 transfectants by calculation and ultrasonic imaging (Figure 8A-8F, p<0.05). The tumors from BTG1 transfectants displayed lower blood supply than the control by Maximum intensity (Imax) of contrast-enhanced ultrasonic imaging (Figure 8G and 8H, p<0.05). HCT-15 and HCT-116 cells showed higher proliferation and lower autophagy than the transfectants, evidenced by Ki-67 and LC-3B immunostaining respectively (Figure 6I). There appeared a higher apoptosis in BTG1 transfectants than the control according to TUNEL assay (Figure 6I).

## DISCUSSION

In the present study, BTG1 protein was mainly localized in the cytoplasm of colorectal mucosal epithelium, infiltrating inflammatory cells, macrophages, lymphoid follicle, adenoma, cancer and hepatocytes. In line with previous reports [18-24], we observed that BTG1 expression was lower in cancer than matched mucosa at the mRNA level, but higher at the protein level. To further confirm these results, the smooth muscle tissues were removed from the colorectal mucosa to avoid muscle BTG1 contamination and LCM was also used to collect normal glands and cancer cells to get rid of stromal or inflammatory cells. Our real-time PCR assays for normal glands and cancer cells achieved similar data with those for colorectal tissues. Thus, the most possible explanation for this paradoxical phenomenon was *BTG1* mRNA destabilization and feedback overexpression of BTG1 protein in colorectal cancer cells. In agreement with the findings in breast, lung, ovarian and hepatocellular cancers [16, 17, 24, 25], our functional experiments showed that BTG1 overexpression suppressed proliferation, cell cycle progression and induced apoptosis, autophagy and differentiation in colorectal cancer cells. Our investigation indicated that promoter methylation can partially be responsible for its down-regulated mRNA expression [18]. In xenograft models, BTG1 overexpression might suppress tumor growth, blood supply and proliferation, and induce apoptosis and autophagy. Taken together, our data suggested that reactive BTG1 overexpression might be involved in colorectal carcinogenesis and inhibit its aggressiveness. Although BTG1 overexpression augmented tube formation and cell migration of endothelial cells [26], in vivo inhibitory effect of BTG1 on blood supply was possibly due to its proliferative suppression and negative modulation of MMPs or VEGF expression [16-20].

During cell cycle, Cyclins E and D1 activate Cdks and play an essential and limiting role in the transition between G_1_ and S phase. Overexpressed p21 and p27 bind to cyclins and cdks, which cause G_1_ arrest [27]. Cyclin B1-Cdk1 is involved in the early events of mitosis and Cdc25B activates Cdc2 entrying into mitosis [28]. In HCT-15 transfectants, BTG1-induced G_2_ arrest might be associated with Cyclin B1 and Cdc25B hypoexpression, while G_1_ progression to p21 and p27 hypoexpression. As for HCT-116 transfectants, G_1_ arrest might be caused by the upregulation of p21 and p27. The higher expression of Cyclin B1 and Cdc2 might result in the low ratio of G_2_-phase cells in HCT-116 transfectants. No matter how high or low in Cyclin E expression, Cyclin D1 and Cdk4 was linked to G_1_ arrest, indicating that the formation of their complexes is not a determinant for G_1_ progression.

BTG1 overexpression induced apoptosis in HCT-15 and HCT-116 cells, but only reduced the mitochondrial potential and increased the cellular senescence in HCT-116 transfectants, suggesting the mechanisms underlying the apoptosis in these two cell lines are different. Thus, we evaluated the apoptosis regulators including Bcl-2, Bcl-xL, Bax, survivin, XIAP, Akt1 and tumor suppressor p53 in BTG1-overexpressing cells. In line with previous studies [20-24, 29, 30], we observed that BTG1 overexpression down-regulated the protein levels of Bcl-2, Bax and up-regulated the expression of p53 in HCT-15 cells. However, HCT-116 transfectants showed the decrease of AIF and XIAP and the elevation of Bcl-2, Bax and p53 proteins. The distinct expression apoptosis-related genes and their encoding proteins might underlie molecular mechanism of the difference in apoptotic alteration of both BTG1-overexpressing cancer cells. Future studies need to be performed for this apoptosis induced by BTG1.

Wnt signaling pathway can inhibit GSK-3-mediated phosphorylation of β-catenin for the nuclear entry of β-catenin, where the interaction of β-catenin with TCF family transcription factors regulates gene expression [31]. Here, we found that ectopic BTG1 expression reduced the mRNA and protein expression of β-catenin and down-regulated its phosphorylation in colorectal cancer cells. It was true for β-catenin-targeting TCF-4 promoter activity. Therefore, we hypothesized that BTG1 overexpression could weaken the β-catenin pathway from transcription via TCF-4 transcriptional factor. Reportedly, activated p38 MAP kinase by phosphorylation at Thr-180 and Tyr-182 has been shown to phosphorylate and activate MAPKAP kinase 2 and to phosphorylate the transcription factors ATF2, Mac and MEF2, finally to modulate the transcriptional expression of inflammatory cytokines such as interleukins [32]. The different changes of interleukins’ expression might be attributed to the differential regulation of phosphor-p38 by BTG1 overexpression in HCT-15 and HCT-116 cells.

Autophagy is a protein-degradation system characterized by the formation of autophagosomes. In the canonical starvation-induced pathway, mTOR kinase regulates autophagy through regulating the components of the ATG13-ULK1-RB1CC1 complex. Subsequently, autophagosome formation is induced by class III PI3K, Beclin-1 and ATG14, finally to promote ATG12-ATG7 conjugation [33]. In the present study, we found that BTG1 overexpression up-regulated the expression of ATG7, ATG14 and Beclin-1 in both colorectal cancer cells. It was suggested that BTG1-induced autophagy was dependent on Beclin-1 and belonged to classical pathway.

Paralleling with their apoptotic induction, the sensitivities of HCT-15 and HCT-116 transfectants to chemotherapeutic agents were different, which implied that the BTG1-mediated sensitization was dependent on cell types and reagents. BCRP and MRP1 proteins act as xenobiotic transporters in multi-drug resistance to chemotherapeutic agents [34]. Both GRP78 and GST-π are involved in the development of chemoresistance [34, 35]. Here, the mRNA expression of the four genes was up-regulated in BTG1 transfectants, suggesting that the correlation between BTG1 expression and drug sensitivity might be not linked to these drug resistance genes.

Up-regulated expression of BTG1 plays an important role in colorectal carcinogenesis as a potential biomarker. BTG1 expression might reverse aggressive phenotypes and be employed as a target of gene therapy for colorectal cancer.

## MATERIALS AND METHODS

### Cell culture

Colorectal cancer cell lines (HCT-15 and HCT-116) were kindly presented by Prof. Miyagi, Clinical Research Institute, Kanagawa Cancer Center, Japan. All cells were cultured in RPMI 1640 medium (HyClone, Logan, UT) supplemented with 10% fetal bovine serum (HyClone, Logan, UT).

### Plasmid construction and transfection

*BTG1* gene was amplified using forward primer: 5’-CCGGAATTCATGCATCCCTTCTACACC-3’, backward primer 5’-GCTCTAGAACCTGATACAGTCATCATAT-3’, and the template cDNA from HCT-15. The PCR products were inserted into pcDNA3.1 (Clontech, USA) between *EcoR*I and *Xba*I, which was directly sequenced. HCT-15 and HCT-116 cells were transfected with pcDNA3.1-BTG1/pcDNA3.1 vector after seeding on dish, selected by G418 with final collection of three monoclones.

### Proliferation assay

The proliferation of different cells was determined by CCK-8. Cells were cultivated in 96-well plates at a density of 2.5×10^3^ cells/well and allowed to adhere. The cell might exposed to paclitaxel (Harbin Pharmaceutical Group Co., Ltd), cistplatin (Hansoh Pharm, DDP), SAHA (Cayman Chem Com, a HDAC inhibitor), MG132 (Enzo, proteosome inhibitor). At different points in time, 10 μL CCK-8 was added into each well of the plates and the plates were measured at 450 nm after a four hours’ incubation.

### Cell cycle analysis

The cells were trypsinized, collected, and fixed in cold 3 mL 75% ethanol. Then, the cells were washed by PBS twice and incubated with 1mL RNase (0.25 mg/mL) at 37°C. Propidium iodide (PI, 50μg/mL, Keygen, China) was added in cell suspension, the cells were incubated at room temperature in the dark. Finally, flow cytometry was employed to examine PI signal.

### Annexin-V-FITC labeling and fluorescence-activated cell sorting analysis (FACS)

Apoptotic cells were determined by FACS analysis after labeling with Annexin-V/PI (Keygen, China). Briefly, cells might be exposed to 0μg/mL, 1.5μg/mL, 3μg/mL of paclitaxel (Harbin Pharmaceutical Group Co., Ltd), 0μM, 5μM, 10μM of cistplatin (Hansoh Pharm, DDP), 0μM, 1.5μM, 3μM of SAHA (Cayman Chem Com, a HDAC inhibitor), 0μM, 0.5μM, 1μM of MG132 (Enzo, proteosome inhibitor) for 48h. The cells were trypsinized, collected and washed by PBS twice. Annexin-V-FITC (5 μl) and 10 μl 50 mg/l PI were added to 500 μl of cell suspension and incubated with cells in the dark for 15 min at room temperature. The cells were analyzed by flow cytometry.

### Mitochondrial membrane potential

The mitochondrial membrane potential was measured according to the protocol of JC-1 Mitochondrial Membrane Potential Assay Kit (Keygen, China). Briefly, the cells were incubated in the incubator for 24h. The cells were collected and washed. JC-1 was added to a final concentration of 1mM with JC-1 monomer (green) as FL1 channel and JC-1 aggregates (red) as FL2 channel.

### Alkaline phosphatase (ALP) activity

Diagnostics ALP reagent (Sigma, USA) was used for ALP activity analysis. The cells were harvested and then subjected the proteins to the determination of ALP activity using multiscan spectrum (Tecan, Switzerland). Protein concentration was determined using Biorad protein assay kit (Biorad, USA). ALP activity was calculated as U/g of protein.

### Transmission electron microscopy

Specimens were immersed in 2% cacodylate-buffered glutaraldehyde. They were then rinsed in cacodylate buffer supplemented with 15% sucrose, post-fixed with 1% phosphate-buffered OsO4 (pH 7.4), dehydrated with alcohol, clarified in propylene oxide, and embedded in Epon using flat molds. Ultrathin sections were made with ultramicrotome (Leica, Germany), stained with uranyl acetate, followed by a saturated solution of bismuth subnitrate and finally examined under a Hitachi electron microscope (Hitachi, Japan).

### β-galactosidase staining

β-galactosidase staining was performed with a senescence-associated β-Galactosidase Staining Kit (Beyotime, China). Cells (5×10^5^) were seeded in 6-well dishes, incubated for 2 days. All cells were washed twice with PBS and fixed with 4% paraformaldehyde for 15 min at room temperature. Then the cells were incubated overnight at 37°C with the working solution containing 0.05 mg/mL X-gal. Finally, the cells were examined under a light inverted microscope (Olympus, Japan).

### Luciferase reporter assay

Cells were seeded in 24-well dishes, The luciferase reporter assay was performed post 48 h of transfection in cells using the Dual-Luciferase^®^Reporter Assay System (Promega, USA) as suggested by the manufacturer. The pGL3-TK was used as a negative control. The Renilla luciferase activity was used as an internal control. TCF-4-mediated gene transcription activity was determined by the ratio of pGL3-OT to pGL3-OF luciferase activity, which was normalized to Renilla luciferase activity of the control plasmid, pRL-TK. TCF-4 promoter activity was determined by the value of pGL-[1306] TCF4-Luc luciferase activity, which was also normalized by Renilla luciferase activity of pRL-TK.

### Immunofluorescence

Cells were grown on glass coverslips until adhesion, fixed with 4 % formaldehyde in PBS for 10 min, and permeabilized with 0.2% Triton X-100/PBS at room temperature. As for paraffin-embedded specimens (4μm), the sections were rehydrated by xylene, subjected to antigen retrieval by irradiating in target retrieval solution (TRS, DAKO, USA) with microwave oven. The sections were blocked by 5% bovine serum albumin (Sigma, USA) and then incubated overnight at 4°C with LC-3B, p38, p-p38, β-catenin and p-β-catenin (Abcam, Cambridge, UK). They were then washed with PBS and incubated with Alexa Fluor 488 IgG (Invitrogen, USA). Nuclei were stained with 1 μg/mL DAPI (Sigma, USA) in the dark. Finally, coverslips were mounted with SlowFade^®^ Gold antifade reagent (invitrogen, USA) and visualized in a laser confocal scanning microscope (Leica, Germany).

### Subjects

Colorectal cancers (n=485), adjacent adenoma (n=109), adjacent non-neoplastic mucosa (n=475), metastatic foci in lymph node metastasis (n=147) and in liver (n=22) were collected from the surgical resection in the Affiliated Hospital, Kanagawa Cancer Center between 1995 and 2007. All tissues were fixed in 10% neutral formalin, embedded in paraffin and cut into at 4 μm. Thirty-six cases of CRCs and paired NNM were collected from the First Affiliated Hospital of China Medical University and frozen in -80°C until protein and RNA extraction by homogenization. None of the patients underwent chemotherapy, radiotherapy or adjuvant before surgery. They all provided written consent for use of tumor tissue for clinical research and the Ethical Committee of our university, Kanagawa Cancer Center and China Medical University approved the research protocol.

### Xenograft models

Locally bred female Balb/c nude (nu/nu) mice were used for implantation at the age of 6-8 weeks. They were maintained under specific pathogen-free conditions, and food and water were supplied ad libitum. Housing and all procedures were performed according to protocols approved by the Committee for Animal Experiments Guidelines on Animal Welfare of Jinzhou Medical University. Subcutaneous xenografts were established by injection of 1× 10^6^ cells per mouse to axilla (n=20 mice/group). After anesthetization and ultrasonic examination, the mice were photographed, and sacrificed. For each tumor, measurements were made using calipers, and tumor volumes were calculated as follows: width^2^× length ×0.52. The part of tumors were subsequently fixed in 4% paraformaldehyde for 24 h, and then embedded in paraffin for following experiments.

### Contrast-enhanced ultrasonic imaging

Ultrasound images of xenograft tumor (n=10/group) were obtained on anaesthetized nude mice using a Philips iU22 (Bothell, WA, USA) ultrasound scanner with the curve-linear array probe C5-2. The imaging parameters were power modulation (PM 3 pulses) transmit frequency 1.7 MHz at low transmit power (mechanical index, 0.06), approximately 7–10 frames per s and one focus well below the level of the target lesion to ensure a more uniform pressure field. At each imaging session, tumor volumes were assessed in fundamental B-mode imaging using calipers. They were calculated using the formula: length × width ×depth× 0.52. During each imaging session, mice were subjected to an intravenous bolus of 0.2 mL of contrast agent (sulfur hexafluoride, SonoVue: Bracco, Italy). Subsequently, color Doppler flow imaging was employed to visualize contrast refilling in tumor. All the experiments were recorded on digital videotapes (Digital Video Recorder Sony, GV-D900E PAL). Time intensity curves and flash replenishment curves were generated in a region of interest including the entire tumor volume using the software Pulse (Bracco Research, Switzerland).

### Laser capture microdissection (LCM) and RNA extract

Eight μm tissue slides were generated and stained by LCM Staining Kit (Ambion) to identify colorectal adenocarcinoma foci versus normal glandular epithelium. LCM was performed using an Arcturus Veritas™ laser capture microdissection system (Life Technologies). Areas of interest were individually captured onto CapSure^®^ Macro LCM caps (Life Technologies, Grand Island, NY). Total RNA was extracted from LCM captured material using Pure Link RNA Mini Kit (Ambion).

### Quantitative RT-PCR

Total RNA was isolated from colorectal cancer and tissues using RNeasy kit (QIAGEN, Germany). The first strand cDNA synthesis was performed using AMV reverse transcriptase and random primer (Takara, Japan). Real-time PCR amplification of cDNA was performed using SYBR Premix Ex Taq™ II kit (Takara, Japan). Standard curves and PCR results were analyzed using ABI7500 software (Applied Biosystems, USA). Primers were shown in Supplementary Table S1. *GAPDH* was used as an internal control.

### Western blot

Proteins were extracted in RIPA lysis buffer by sonication. Lysates were measured for protein concentration using the Bradford protein assay. Denatured proteins were separated on an SDS-polyacrylamide gel and transferred to Hybond membranes, which were then blocked overnight in 5% skim milk/TBST (tris-buffered saline Tween-20). For immunoblotting, membrane was incubated with the primary antibodies (Supplementary Table S2). Then, it was rinsed twice by TBST and incubated with IgG-conjugated to horseradish peroxidase (DAKO, Denmark). Densitometry quantification was performed with an internal control of β-actin or GAPDH using Scion Image software. Additionally, the expression level of the control was considered as “1”.

### Tissue microarray and immunohistochemistry

Under the guidance of HE sections, a two mm-in-diameter human tissue core per donor block was punched out and transferred to a recipient block with a maximum of 48 cores using a Tissue Microarrayer (AZUMAYA). The immunohistochemical procedures were performed as described previously [36]. The specimens (4μm) were incubated with the antibody for against BTG1 (Cell signaling technology, USA), ki-67 (Abcam, Cambridge, UK) or LC-3B (Cell Signal). As for immunofluorescence of LC-3B, Alexa Fluor 488 IgG (Invitrogen) was used as a secondary antibody and DAPI (Sigma) as nuclear staining. Finally, coverslips were mounted with SlowFade^®^ Gold reagent (invitrogen) and observed under laser confocal scanning microscope. Omission of the primary antibody was used as a negative control.

As indicated in Figures 7 and 8, BTG1 protein was positively localized in the cytoplasm, while ki-67 in the nucleus. To semiquantify BTG1 expression, one hundred cells were randomly selected and counted from 5 representative fields blindly by both independent observers. The expression positivity was graded and counted as follows: 0 =negative; 1 = 1-50%; 2 = 51-74%; 3 ≥75%. The staining intensity score was graded as follows: 1 = weak; 2 = intermediate; and 3 = strong. The scores for BTG1 positivity and staining intensity were multiplied to obtain a final score, which determines their expression as (- = 0; + = 1-2; ++ = 3-5; +++ = 6-9).

### Terminal digoxigenin-labeled dUTP nick-end labeling (TUNEL)

Cell apoptosis was assessed using TUNEL, which is based on the specific binding O-TdT to the 3-OH ends of DNA, ensuring the synthesis of a polydeoxynucleotide polymer. For this purpose, ApopTag Plus Peroxidase In Situ Apoptosis Detection Kit (Chemicon) was employed according to the recommendation. Omission of the working strength TdT enzyme was considered as a negative control.

### Statistical analysis

Results are representative of 3 different experiments, and data are expressed as mean ± standard deviation. Statistical evaluation was performed using Spearman's correlation test to analyze the rank data, and Mann-Whitney U to differentiate the means of different groups. SPSS 10.0 software was applied to analyze all data and *p* <0.05 was considered statistically significant.

## SUPPLEMENTARY MATERIALS FIGURES AND TABLES


