# Supplementary material for: BTG1 might be employed as a biomarker for carcinogenesis and a target for gene therapy in colorectal cancers
Source: Oncotarget. 2016 Jul 18;8(5):7502–20. doi: 10.18632/oncotarget.10649 (PMC5352338; doi:10.18632/oncotarget.10649)
Supplement: Supplementary file 1 [file oncotarget-08-7502-s001.pdf]

BTG1 might be employed as a biomarker for carcinogenesis and a target for gene therapy in colorectal cancers

SUPPLEMENTARY TABLES

Supplementary Table S1: The primers used in Real-time RT-PCR

Names	Primer's sequence	Distribution	AT (°C)	Product size(bp)	Extension time(s)
Cyclin B1	F: 5'-GTTATGCAGCACCTG-3'<br>R: 5'-CTTGGCTAAATCTTGAACT-3'	NM_001088590<br>1388-1537	60	150	34
CyclinE1	F: 5'-GGATGTTGACTGCCTTGA-3'<br>R: 5'-CGCACCACTGATACCCT-3'	BA000005<br>1044-1150	60	107	34
Cdc2	F: 5' –GGGCACTCCCAATAA-3'<br>R: 5'-GATGCTAGGCTTCCTG-3'	XM_572099<br>631-723	60	93	34
Bcl-2	F: 5'-GCCTTCTTTGAGTTCGGTGGG-3'<br>R: 5'-TGTGCAGGTGCCGGTTCAG-3'	DQ926871<br>938-1052	60	115	34
Bax	F: 5'-GATTGCCGCCGTGGAC-3'<br>R: 5'-GCCCCAGTTGAAGTTGC-3'	DQ926869<br>306-393	60	88	34
Bcl-xL	F: 5'-GGCAACCCATCCTGGCACCT-3'<br>R:5'-AACTCGTCGCCTGCCTCCCT-3'	AY263145<br>118-276	60	159	34
Akt1	F: 5'-TCTTTGCCGGTATCGTGT-3'<br>R: 5'-TGTCATCTTGGTCAGGTGGT-3'	F283818S13<br>1559-1708	60	150	34
P21	F: 5'-ACTGTCTTGTACCCTTGTGCC-3'<br>R: 5'-AAATCTGTCATGCTGGTCTGC-3'	XM_003950827<br>572-679	60	108	34
P27	F: 5'-GGCTCCGGCTAACTCTGA-3'<br>R: 5'-TTCTTCTGTTCTGTTGGCTCTT-3'	XM_522347<br>1081-1237	60	157	34
GRP78	F: 5'-GTTCTTGCCGTTCAAGGTGG-3'<br>R:5'-TGGTACAGTAACAACTGCATG-3'	FJ436356<br>600-780	60	181	34
BCRP	F: 5'-GACAGCTTCCAATGACCTGAA-3'<br>R: 5'-CAGGATGGCGTTGAGACC-3'	XM_005263356<br>285-456	60	172	34
MRP1	F: 5'-TTTCAGAACACGGTCCTCG-3'<br>R: 5'-TGGGCTGACCAGAAACACT-3'	XM_005255327<br>190-423	60	234	34
GSTπ	F: 5'-CGGGCAAGGATGACTATGTGA-3'<br>R: 5'-GGGCTAGGACCTCATGGATCA-3'	XM_001152516<br>585-746	60	162	34
β-catenin	F: 5'-GCTTGGAATGAGACTGCTGA-3'<br>R: 5'-CTGGCCATATCCACCAGAGT-3'	X87838<br>2221-2334	60	114	34
cyclinD1	F: 5'-TGCCACAGATGTGAAGTTCATT-3'<br>R: 5'-CAGTCCGGGTCACACTTGAT-3'	NG_000002<br>776-937	60	162	34
Survivin	F: 5'-TTCTCAAGGACCACCGCATC-3'<br>R: 5'-AGCCTTCCAGCTCCTTGAAG-3'	DQ508252<br>159-320	60	162	34
IL-2	F: 5'-GACTTTACTGCTGGATTT-3'<br>R: 5'-ATTGCTGATTAAGTCCCT-3'	NM_000586.3<br>160-379	60	220	34
IL-4	F: 5'-CAGTTCTACAGCCACCAT-3'<br>R: 5'-CTGGTTGGCTTCCTTCAC-3'	NM_172348.2<br>249-407	60	159	34
IL-17	F: 5'-GAAGGCAGGAATCACAAT-3'<br>R:5'-ATCGGTTGTAGTAATCTG-3'	NM_002190.2<br>108-253	60	146	34
GAPDH	F: 5'-CAATGACCCCTTCATTGACC-3'<br>R: 5'-TGGAAGATGGTGATGGGATT-3'	NM_002046.3<br>201-335	60	135	34

AT=annealing temperature.

Supplementary Table S2: Antibodies' used in Western blot

Names	Source	Company
Cyclin B1 (GNS1)	Mouse	Santa Cruz Biotech. Inc.
Cyclin D1 (H-295)	Rabbit	Santa Cruz Biotech. Inc.
CyclinE1 (HE12)	Rabbit	Santa Cruz Biotech. Inc.
Cdc2 p34 (B-6)	Mouse	Santa Cruz Biotech. Inc.
Cdk4 (C-22)	Rabbit	Santa Cruz Biotech. Inc.
Cdc25B (C-20)	Rabbit	Santa Cruz Biotech. Inc.
P53 (FL-393)	Rabbit	Santa Cruz Biotech. Inc.
Bcl-2 (C 21)	Rabbit	Santa Cruz Biotech. Inc.
Bax (B-9)	Mouse	Santa Cruz Biotech. Inc.
AIF(E-1)	Mouse	Santa Cruz Biotech. Inc.
XIAP(H-202)	Rabbit	Santa Cruz Biotech. Inc.
Atg14	Rabbit	Abcam
Beclin 1	Rabbit	Abcam
Atg7 (N-20)	Goat	Santa Cruz Biotech. Inc.
β-catenin (C-18)	Goat	Santa Cruz Biotech. Inc.
Phospho-β-catenin (Y142)	Rabbit	Abcam
P38α/β(H-147)	Rabbit	Santa Cruz Biotech. Inc.
Phospho-p38α/β(Thr180/Tyr182)	Rabbit	Santa Cruz Biotech. Inc.
Ki67	Rabbit	Abcam
LC3B	Rabbit	Cell signaling technology
BTG1	Rabbit	Proteintech
β-actin (C-4)	Mouse	Santa Cruz Biotech. Inc.
GAPDH	Mouse	Santa Cruz Biotech. Inc.
